# Vitronectin Modulates Plasma Aβ Oligomerization Propensity Within Altered Albumin Interactome Networks in Alzheimer’s Disease

**DOI:** 10.3390/ijms27135744

**Published:** 2026-06-25

**Authors:** Hojin Kang, Hongju Kim, Woo Jung Kim, Leon French, Hyunjung Oh, Seong Soo A. An

**Affiliations:** 1 PeopleBio Inc., 6th Floor, PDC C-dong, 242 Pangyo-ro, Bundang-gu, Seongnam-si 13487, Republic of Korea; 2 Department of Psychiatry, Yonsei University College of Medicine, Yongin Severance Hospital, 363 Dongbaekjukjeon-daero, Giheung-gu, Yongin-si 16995, Republic of Korea; 3 Institute of Behavioral Sciences in Medicine, Yonsei University College of Medicine, 50-1 Yonsei-ro, Seodaemun-gu, Seoul 03722, Republic of Korea; 4 Yonsei Graduate Program in Cognitive Science, Yonsei University, 50 Yonsei-ro, Seodaemun-gu, Seoul 03722, Republic of Korea; 5 Physiology Department and Donnelly Centre for Cellular and Biomolecular Research, University of Toronto, 160 College St, Toronto, ON M5S 3E1, Canada; 6 Bionano Application Research Center, Gachon University, 1342 Seongnam-daero, Sujeong-gu, Seongnam-si 13120, Republic of Korea; 7 Department of Bionano Technology, Gachon University, 1342 Seongnam-daero, Sujeong-gu, Seongnam-si 13120, Republic of Korea

**Keywords:** Aβ oligomerization tendency, albumin interactome, vitronectin

## Abstract

Amyloid beta (Aβ) oligomers are key mediators of synaptic dysfunction and neural circuit impairment in Alzheimer’s disease (AD). While plasma Aβ oligomerization propensity (OAβ) correlates with cerebral amyloid pathology and cognitive decline, the systemic modulators of OAβ remain poorly understood. In this study, we identified the albumin interactome (albumin and its associated proteins) as a critical regulator of OAβ. Selective depletion of the albumin interactome from plasma eliminated the OAβ difference between amyloid PET (A-PET)- and A-PET+ individuals. Proteomic analysis revealed widespread network alterations within the albumin interactome of A-PET+ individuals. Notably, vitronectin (VTN) was identified as a key hub protein that was significantly reduced in A-PET+ individuals. Functional assays and in silico modeling demonstrated that VTN directly bound to Aβ and inhibited its oligomerization. Additionally, plasma VTN levels distinguished A-PET status. These findings suggest that systemic changes in the albumin interactome, particularly the reduction in VTN, are associated with dysregulated Aβ dynamics in plasma. Our results provide novel insights into systemic mechanisms underlying AD pathology and identify VTN as a potential peripheral modulator and biomarker of cerebral amyloid pathology.

## 1. Introduction

Alzheimer’s disease (AD) is the most prevalent neurodegenerative disease characterized by progressive cognitive decline and memory impairment. Synaptic loss has been identified as the strongest correlate of cognitive decline in AD [1,2,3]. Advanced molecular imaging techniques, such as positron emission tomography (PET) targeting synaptic vesicle glycoprotein 2A (SV2A) [4], have demonstrated a significant reduction in hippocampal synaptic density (approximately 40%) in individuals with AD compared to cognitively unimpaired controls [4]. Soluble amyloid-β (Aβ) oligomers are widely recognized as major neurotoxic species implicated in AD pathogenesis, as they disrupt synaptic function, neuronal excitability, and network connectivity in the brain [5,6,7,8].

Although Aβ accumulation is believed to commence 15 to 20 years prior to clinical onset [5], direct quantification of Aβ oligomers in blood remains challenging due to their extremely low abundance and transient nature [9]. To assess AD-related molecular changes using blood samples, we recently developed a blood-based diagnostic assay that measures the propensity for Aβ oligomerization (OAβ) by spiking plasma with synthetic Aβ and detecting oligomeric Aβ (Multimer Detection System (MDS)-OAβ). Multiple clinical studies have shown that OAβ effectively distinguished amyloid PET-positive (A-PET+) individuals and correlated with key AD biomarkers, including amyloid pathology, cognitive function, and cerebrospinal fluid (CSF) biomarkers [9,10,11,12,13,14,15,16,17,18]. Additionally, OAβ-associated proteomic signatures were linked to immune responses, impaired cellular metabolism, and accelerated muscle aging, indicating that systemic biological environments may influence the propensity for Aβ oligomerization in the blood [19]. Notably, OAβ is also associated with white matter integrity and structural brain changes [15], suggesting that systemic factors regulating OAβ may reflect biological processes underlying early neural circuit dysfunction in AD.

Recent evidence suggests that peripheral blood components are not merely passive byproducts of AD but rather active participants in systemic molecular pathways associated with neural circuit integrity. Transfusion of blood from older mice into young mice has been shown to reduce hippocampal neurogenesis and cognitive function [20,21,22], exacerbate amyloidosis and neuroinflammation [23], and disrupt proteins associated with synaptic function in young AD model mice [24]. In contrast, administration of young blood to older AD model mice reduced brain Aβ burden and improved memory [25]. Collectively, these findings demonstrate that circulating blood factors can modulate AD-related pathological processes through systemic molecular interactions. Human serum albumin, the most abundant plasma protein, has been identified as a major carrier of Aβ in the blood. Approximately 90–95% of circulating Aβ is bound to albumin or albumin-associated components [26,27]. Albumin interacts with a range of plasma proteins, collectively referred to as the albumin interactome [28], which may regulate Aβ dynamics in circulation. Within the albumin interactome, vitronectin (VTN), a multifunctional glycoprotein [29], has emerged as a key candidate. VTN inhibited Aβ fibrillization [30] and was present in amyloid plaques in the brains of AD patients [31]. However, the role of VTN as a peripheral modulator of Aβ oligomerization remains unknown.

This study examined the role of albumin interactome in regulating OAβ levels. To identify the proteins involved, the albumin interactome was fractionated using size-exclusion chromatography (SEC) and analyzed by liquid chromatography–tandem mass spectrometry (LC-MS/MS). Protein abundance was compared between A-PET− and A-PET+ groups, and group-specific protein co-expression networks were constructed. The results revealed global alterations in albumin interactome protein expression in A-PET+ individuals and identified VTN as a key regulator of OAβ. These findings support the biological significance of systemic plasma proteomic environments in modulating Aβ oligomerization propensity and suggest potential links between peripheral protein dynamics and early neural circuit dysfunction in AD.

## 2. Results

### 2.1. Plasma Amyloid Beta Oligomerization Propensity (OAβ) Was Elevated in Individuals with Cerebral Amyloid Pathology

**Figure 1:**
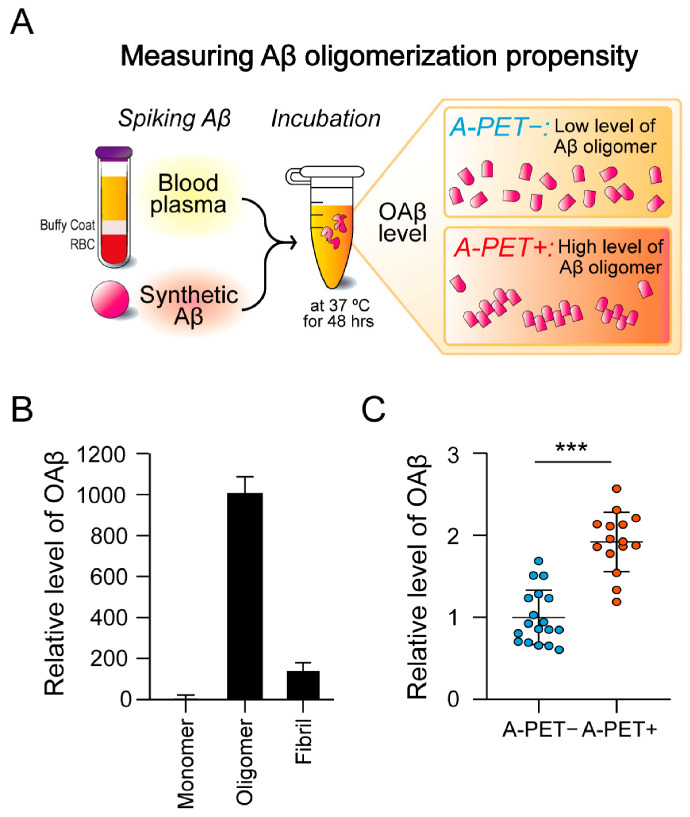
Amyloid beta oligomerization propensity (OAβ) is significantly elevated in the plasma of amyloid PET(A-PET)-positive individuals (A-PET+). (**A**) Concept of the Multimer Detection System-OAβ (MDS-OAβ) system. Synthetic Aβ is spiked in plasma and incubated for 48 h. The level of oligomerized Aβ is measured by sandwich enzyme-linked immunosorbent assay (ELISA). (**B**) The relative levels of OAβ in different Aβ species (monomer, oligomer, fibril); *n* = 3 per group. (**C**) The relative levels of OAβ in A-PET− and + individuals (A-PET−, *n* = 18; A-PET+, *n* = 15). Statistical significance was determined by unpaired Student’s *t*-test. Data are expressed as mean ± SEM. *** *p* < 0.001.

To evaluate Aβ oligomerization propensity in blood plasma, synthetic Aβ was spiked into plasma from individuals with or without cerebral amyloid pathology. Plasma OAβ levels were measured using MDS-OAβ (Figure 1A), which selectively detects oligomeric Aβ with negligible cross-reactivity to monomeric or fibrillar forms (Figure 1B). Upon Aβ introduction, plasma from A-PET+ subjects exhibited 1.9-fold higher OAβ levels compared to A-PET− subjects (A-PET−, *n* = 18; A-PET+, *n* = 15) (*p* < 0.001, Figure 1C). These results indicate that plasma OAβ is elevated in individuals with cerebral amyloid pathology and suggest that OAβ may reflect systemic conditions associated with early brain changes in AD.

### 2.2. Albumin-Associated Plasma Proteins Regulate Plasma Aβ Oligomerization Propensity

**Figure 2:**
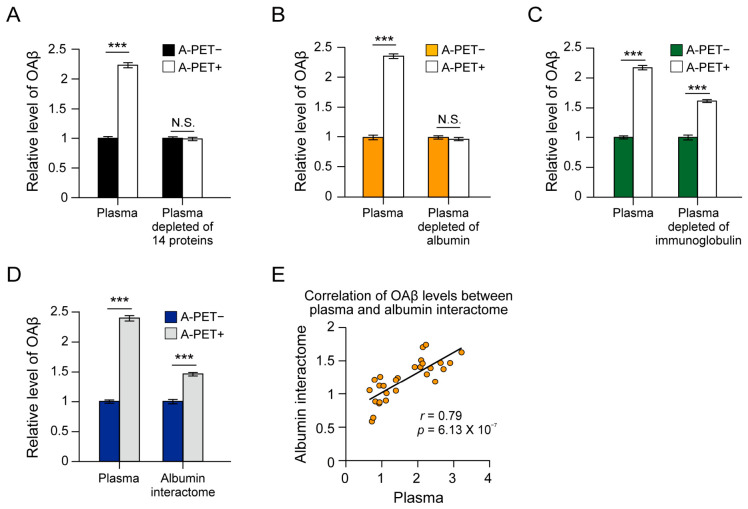
The albumin interactome is necessary to distinguish OAβ levels by A-PET status. (**A**) The relative levels of OAβ in A-PET-negative (A-PET−) and + individuals’ plasma with or without the depletion of 14 abundant proteins (A-PET−, *n* = 15; A-PET+, *n* = 13). (**B**) The relative levels of OAβ in A-PET− and + individuals’ plasma with or without the depletion of albumin (A-PET−, *n* = 15; A-PET+, *n* = 13). (**C**) The relative levels of OAβ in A-PET− and + individuals’ plasma with or without the depletion of immunoglobulin (A-PET−, *n* = 15; A-PET+, *n* = 13). (**D**) The relative levels of OAβ in the purified albumin interactome of A-PET− and + individuals (A-PET−, *n* = 15; A-PET+, *n* = 13). (**E**) Correlation of OAβ levels between plasma and the purified albumin interactome (A-PET−, *n* = 15; A-PET+, *n* = 13). Statistical significance was determined by unpaired Student’s *t*-test. Data are expressed as mean ± SEM. *** *p* < 0.001. N.S., not significant.

To identify plasma components influencing OAβ levels, we selectively depleted major plasma proteins. OAβ levels were 2.2-fold higher in A-PET+ individuals compared to A-PET− controls (A-PET−, *n* = 15; A-PET+, *n* = 13). Depletion of the 14 most abundant plasma proteins completely abolished this difference (Figure 2A). We then depleted albumin, the most abundant plasma protein [32], and observed the elimination of the group difference (Figure 2B). In contrast, depletion of immunoglobulins, the second most abundant protein class, slightly reduced OAβ levels in the A-PET+ group, but A-PET+ plasma samples still showed a 1.6-fold higher OAβ level compared to A-PET− samples (*p* < 0.001, Figure 2C). These results suggest that albumin and its associated binding proteins, collectively termed the albumin interactome, play a crucial role in modulating OAβ. Indeed, the purified albumin interactome from A-PET+ individuals showed a 1.5-fold increase in OAβ levels compared to A-PET− controls (*p* < 0.001, Figure 2D). A strong correlation was observed between plasma OAβ levels and paired albumin interactome samples (r = 0.79; Figure 2E). Collectively, these findings indicate that the albumin interactome is a significant determinant of OAβ levels in plasma.

### 2.3. Size-Exclusion Chromatography (SEC) Revealed a Critical Protein Fraction of the Albumin Interactome

**Figure 3:**
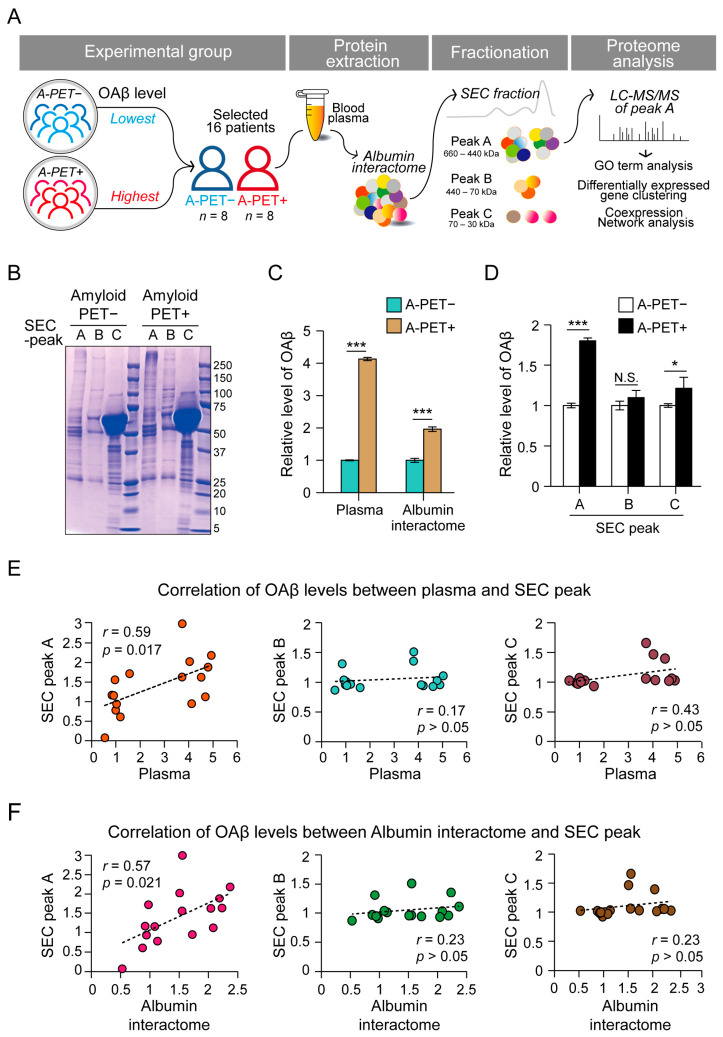
The high-molecular-weight fraction (Peak A) of albumin interactome is responsible for indicating OAβ levels by A-PET status. (**A**) Experimental process for analyzing the proteome of Peak A. (**B**) Expression patterns of Peaks A, B, and C by Coomassie staining. (**C**) The relative levels of OAβ in the purified albumin interactome of A-PET− and + individuals; *n* = 8 per group. (**D**) The relative levels of OAβ in Peaks A, B, and C of A-PET− and + individuals; *n* = 8 per group. (**E**) Correlation of OAβ levels between Peaks A (**left panel**), B (**middle panel**), C (**right panel**), and plasma; *n* = 8 per group. (**F**) Correlation of OAβ levels between Peaks A (**left panel**), B (**middle panel**), C (**right panel**), and albumin interactome; *n* = 8 per group. Statistical significance was determined by unpaired Student’s *t*-test. Data are expressed as mean ± SEM. * *p* < 0.05, *** *p* < 0.001. N.S., not significant.

To identify the specific fraction within the albumin interactome that modulates OAβ levels, SEC was performed on albumin interactome samples from A-PET− and + individuals (*n* = 8 per group). SEC is a well-established method for separating proteins by molecular weight while preserving native protein complexes [28]. To maximize contrast, we selected individuals with the highest and lowest OAβ levels, performed SEC, and measured OAβ levels in each fraction (Figure 3A). SEC fractionation produced three major peaks (A, B, and C), and each fraction exhibited a distinct Coomassie staining pattern (Figure 3B, Supplementary Figure S1A,B). A prominent albumin band was observed in Peak C (Figure 3B). OAβ levels in plasma and albumin interactome were increased in A-PET+ individuals by 4.1-fold and 2.0-fold, respectively (*p* < 0.001, Figure 3C). Among the three fractions, Peak A exhibited the most significant increase, with a 1.8-fold elevation in OAβ levels in A-PET+ samples (*p* < 0.001). Peak B did not show a significant difference, and Peak C showed a 1.2-fold increase (*p* < 0.05) (Figure 3D). Furthermore, the levels of OAβ in Peak A were positively correlated with those in plasma (r = 0.59, Figure 3E) and the total albumin interactome (r = 0.57, Figure 3F). No such correlation was observed in Peaks B or C, suggesting that the proteins within Peak A represent a major plasma component associated with OAβ regulation.

### 2.4. Proteomic and Network Alterations in the OAβ-Associated Albumin Interactome Fraction

**Figure 4:**
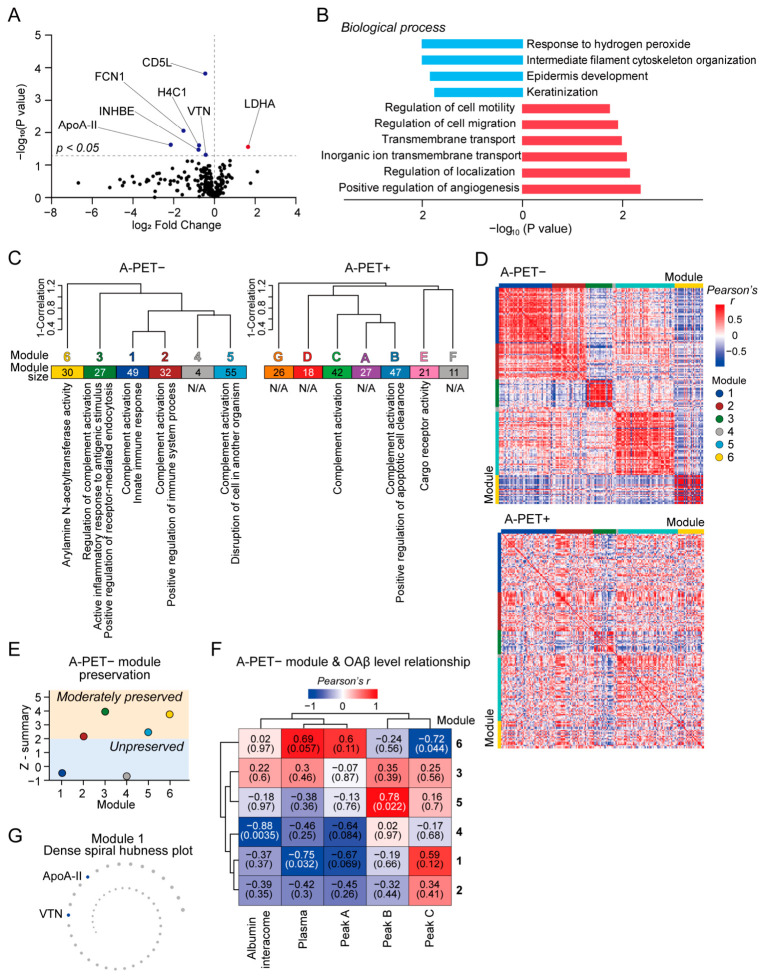
Global proteomic alterations in Peak A of A-PET+ individuals. (**A**) Volcano plot of differentially expressed proteins in A-PET− and +; *n* = 8 per group. (**B**) Gene Ontology (GO) analysis of differentially expressed proteins in A-PET+ compared to A-PET−. (**C**) Co-expression network module clustering dendrogram. Different colors represent different modules (blue: module 1; brown: module 2; green: module 3; grey: module 4; turquoise: module 5; yellow: module 6). (**D**) Heatmap by co-expression network module of A-PET−. (**E**) A-PET− Module preservation score in A-PET+. (**F**) Module–trait relationship between A-PET− module and OAβ level, Pearson’s r (*p*-value). (**G**) Hubness plot of A-PET− module 1. N/A, not available.

To identify specific proteins within Peak A that contribute to differences in OAβ levels, we performed label-free LC–MS/MS analysis. A total of 369 proteins were identified (excluding single-peptide hits, potential contaminants, and proteins lacking abundance data), and 357 proteins were common to both groups. Differential expression analysis showed that lactate dehydrogenase A (LDHA) was significantly upregulated in A-PET+ samples, while six proteins, including CD5 antigen-like (CD5L), ficolin-1 (FCN1), apolipoprotein A-II (Apo A-II), histone H4 (H4C1), inhibin beta E chain (INHBE), and vitronectin (VTN), were significantly downregulated (Figure 4A, Supplementary Table S5). Gene Ontology (GO) analysis revealed enrichment of downregulated proteins in groups such as response to hydrogen peroxide and intermediate filament organization, and enrichment of upregulated proteins in processes related to localization and angiogenesis (Figure 4B, Supplementary Table S6).

To investigate systemic proteome alterations associated with A-PET status, weighted gene co-expression network analysis (WGCNA) was performed (Supplementary Table S7). In the A-PET− group, 197 proteins were assigned to six co-expression modules, whereas in the A-PET+ group, 192 proteins were reorganized into seven distinct modules (Figure 4C, Supplementary Figure S2A). The overall network density was tighter in the A-PET− group (mean density: 0.0639) than in the A-PET+ group (mean density: 0.0427). GO analysis revealed that four of the seven modules of the A-PET+ group lacked enrichment for distinct biological processes (Figure 4C), further indicating disorganization. Heatmap visualization and network topology confirmed that the module integrity of the A-PET− was not preserved in A-PET+ (Figure 4D, Supplementary Figure S2B). Among all modules, module 1 exhibited the most tremendous loss of preservation (Figure 4E). Notably, the module 1 eigengene of the A-PET− group showed the highest correlation with OAβ levels in both plasma (*p* = 0.032) and Peak A (*p* = 0.069) (Figure 4F). Notably, two of the differentially expressed proteins, Apo A-II and VTN, were included in module 1 and showed strong hubness (Figure 4G, Supplementary Table S8). These findings suggest that elevated OAβ is associated not only with changes in individual proteins but also with broader reorganization of systemic protein interaction networks.

### 2.5. Validation of Differentially Expressed Proteins by Enzyme-Linked Immunosorbent Assay (ELISA)

To validate the proteomic findings, we focused on CD5L, Apo A-II, and VTN. CD5L exhibited the most significant alteration, whereas Apo A-II and VTN were identified as hub proteins of module 1. Expression levels of these proteins were assessed using ELISA (Enzyme-Linked Immunosorbent Assay; *n* = 8 per group).

**Figure 5:**
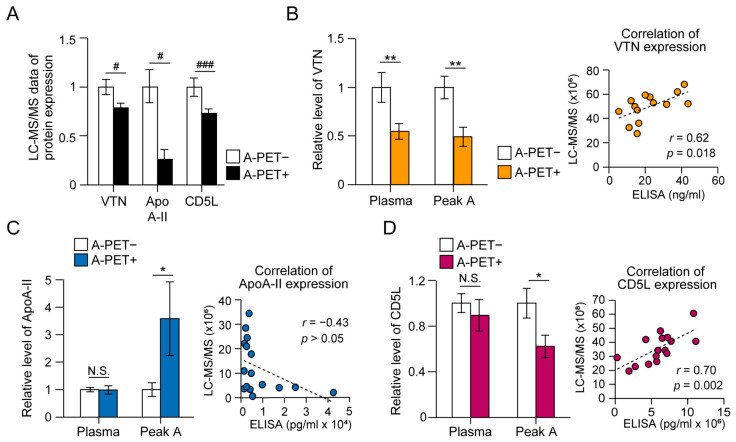
Validation of the expression levels of AD-related proteins in plasma and Peak A. (**A**) Expression levels of vitronectin (VTN), apolipoprotein A-II (Apo A-II), and CD5 antigen-like (CD5L) by liquid chromatography–tandem mass spectrometry (LC-MS/MS) analysis; *n* = 8 per group. (**B**) The relative level of VTN in plasma and Peak A of A-PET− and + individuals, as determined by ELISA; *n* = 7 per group. (**C**) The relative level of Apo A-II in plasma and Peak A of A-PET− and + individuals, as determined by ELISA; *n* = 8 per group. (**D**) The relative level of CD5L in plasma and Peak A of A-PET− and + individuals, as determined by ELISA; *n* = 8 per group. Statistical significance was determined by paired Student’s *t*-test (**A**) or unpaired Student’s *t*-test (**B**–**D**). Data are expressed as mean ± SEM. # *p* < 0.05, ### *p* < 0.001, * *p* < 0.05, ** *p* < 0.01. N.S., not significant.

LC–MS/MS analysis indicated that all three proteins were less abundant in A-PET+ samples (Figure 5A). However, only VTN demonstrated a consistent 50% reduction in both plasma and Peak A (*p* < 0.01, Figure 5B, left panel). A positive correlation was also observed between LC-MS/MS and ELISA measurements for VTN in Peak A (r = 0.62, Figure 5B, right panel). In contrast, Apo A-II levels did not decrease in Peak A from A-PET+ samples (Figure 5C, left panel), and no correlation was observed between LC-MS/MS and ELISA results (Figure 5C, right panel). CD5L levels were reduced in Peak A from A-PET+ samples (*p* < 0.05) but remained unchanged in plasma (Figure 5D, left panel). CD5L in Peak A also showed a positive correlation between LC-MS/MS and ELISA measurements (r = 0.70, Figure 5D, right panel). Together, these findings identified VTN as a robust candidate connecting systemic proteomic alterations with OAβ modulation.

### 2.6. Vitronectin (VTN) Suppresses Aβ Aggregation and Modulates Plasma OAβ

**Figure 6:**
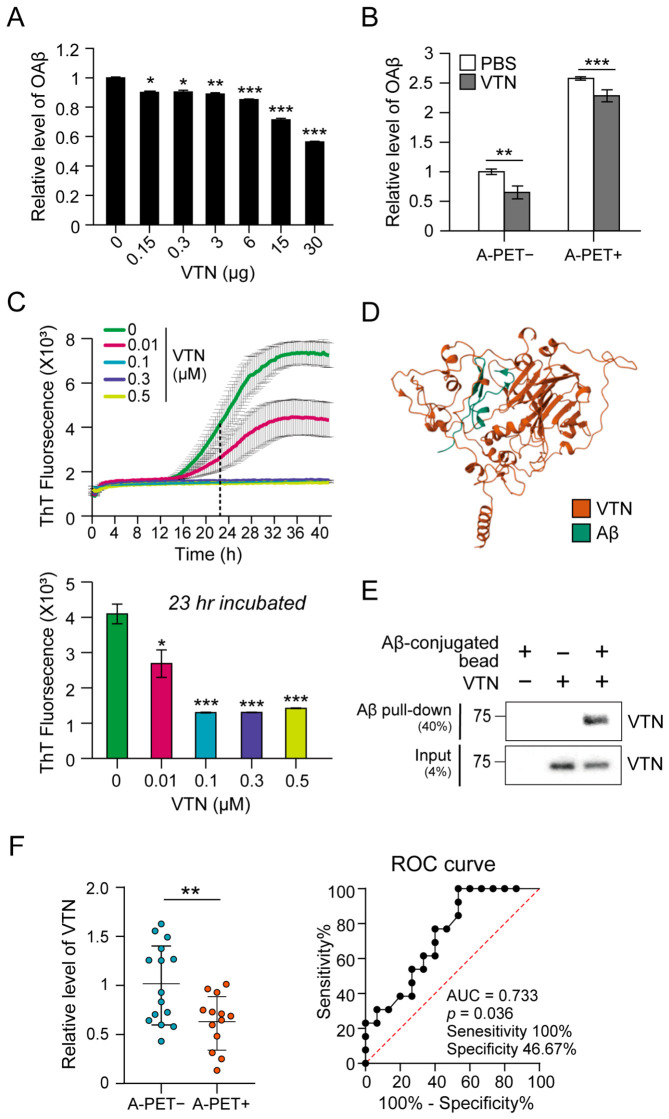
VTN represses Aβ oligomerization in plasma. (**A**) The relative level of OAβ with indicated amount of VTN introduction (0.15, 0.3, 3, 6, 15, 30 μg); *n* = 3. (**B**) The relative level of OAβ in the plasma of A-PET− and + individuals with or without 15 μg of VTN; *n* = 3 per group. (**C**) In vitro fibrillization analysis of Aβ with indicated levels of VTN addition (0.01, 0.1, 0.3, 0.5 μM) using Thioflavin T (ThT) fluorescence (**upper panel**), ThT fluorescence after 23 h incubation (**lower panel**); *n* = 3. (**D**) The Protein–protein interaction model of VTN and Aβ. (**E**) In vitro binding assay of Aβ and VTN. (**F**) The relative level of VTN in plasma, using ELISA (**left panel**). The receiver operating characteristic (ROC) curve (**right panel**) for differentiating A-PET status with plasma VTN levels (Area under the curve (AUC) = 0.733, *p* = 0.036, 100% sensitivity and 46.67% specificity) (A-PET−, *n* = 15; A-PET+, *n* = 13). Statistical significance was determined by One-way ANOVA (**A**,**C**), Two-way ANOVA (**B**), followed by Tukey’s post hoc test, and an unpaired *t*-test (**F**). Data are expressed as mean ± SEM. * *p* < 0.05, ** *p* < 0.01, *** *p* < 0.001.

We next investigated whether CD5L or VTN could directly modulate Aβ oligomerization propensity in plasma. The addition of recombinant CD5L did not alter OAβ levels (Supplementary Figure S3). In contrast, exogenous VTN significantly reduced OAβ levels in a dose-dependent manner, with reductions ranging from 10 to 44%. Administration of 3 µg of VTN, corresponding to the physiological concentration in plasma (3 µg/10 µL [33]), resulted in an 11% decrease in OAβ levels (Figure 6A). This reduction was observed in both A-PET− and A-PET+ plasma samples (Figure 6B). ThT assays demonstrated that VTN suppressed Aβ fibrillization by 68% at 0.1 µM (Figure 6C, upper and lower panels). In silico docking analysis predicted a high-affinity interaction between VTN and Aβ (Aggregate Score: 0.9007, Global PDE: 0.7154) (Figure 6D), and co-immunoprecipitation confirmed direct binding between the two proteins (Figure 6E).

To assess whether VTN can differentiate A-PET status, we measured plasma VTN concentrations in 28 individuals (A-PET−, *n* = 15; A-PET+, *n* = 13). VTN expression was decreased by 39% in the A-PET+ group (*p* < 0.01, Figure 6F, left panel) and distinguished OAβ/A-PET status, with an area under the curve (AUC) of 0.733, a sensitivity of 100%, and a specificity of 46.7% (*p* = 0.036, Figure 6F, right panel). Together, these findings suggest that plasma VTN acts as a peripheral modulator of Aβ aggregation and may reflect systemic molecular states associated with early AD pathology.

## 3. Discussion

In this study, we investigated the influence of the blood plasma proteome on Aβ oligomerization propensity in AD. The albumin interactome was identified as a key modulator of OAβ levels. Depletion of the albumin interactome abolished the plasma’s ability to differentiate A-PET+ from A-PET− individuals. Proteomic profiling of the high-molecular-weight albumin interactome fraction (Peak A) revealed global proteome changes and reorganization of co-expression network modules in the A-PET+ group. The hub protein VTN was significantly decreased in A-PET+ samples. The addition of exogenous VTN reduced OAβ levels and suppressed Aβ fibrillization. Furthermore, plasma VTN levels could differentiate A-PET positivity, highlighting its potential as both a modulatory and diagnostic marker.

As the most abundant general transport protein in plasma, albumin interacts with a wide range of circulating molecules [28,34,35,36]. Our findings demonstrate that the albumin interactome plays a crucial role in distinguishing A-PET+ individuals from A-PET− individuals based on their propensity for Aβ oligomerization (Figure 2). To further characterize its functional components, the plasma albumin interactome was fractionated using SEC. A specific high-molecular-weight fraction, Peak A, was identified as the main contributor to OAβ (Figure 3). Proteomic analysis of Peak A revealed that the A-PET+ group exhibits systemic alterations associated with Aβ pathology, including a decreased response to hydrogen peroxide. This observation is consistent with our previous plasma proteomic study, which showed that elevated OAβ was associated with systemic immune activation, metabolic dysfunction, and accelerated organ-aging signatures [19], indicating that OAβ reflects broad peripheral biological alterations linked to early AD pathology. Several proteins differentially expressed in Peak A among A-PET+ individuals indicate systemic alterations in metabolism, immunity, and lipid homeostasis, all of which are closely associated with AD pathophysiology and accelerated aging (Figure 4). The upregulation of LDHA suggests a metabolic change toward increased lactate production. Elevated lactate levels in both brain and plasma were associated with brain aging [37], which was strongly linked to AD [38]. This aging-related profile was further supported by the downregulation of H4C1, reflecting the natural reduction in histone biosynthesis during aging [39]. Additionally, the reduction in Apo A-II, a key apolipoprotein in high-density lipoprotein (HDL) particles, indicates dysregulated lipid metabolism, which affects Aβ aggregation and clearance [40,41]. In contrast, key immune-related proteins, such as CD5L, FCN1, and INHBE, were significantly downregulated. As CD5L is implicated in cerebral amyloid angiopathy [42] and FCN1 activates the complement pathway [43], their reduction suggests a compromised innate immune environment and impaired Aβ clearance mechanisms. The downregulation of INHBE, a member of the TGF-β (Transforming Growth Factor-Beta) superfamily, in A-PET+ individuals may reflect exhaustion of systemic anti-inflammatory TGF-β signaling [44]. Notably, VTN was significantly reduced and served as a central modulator within this altered network, as discussed in subsequent sections.

Beyond these individual changes, co-expression network analysis revealed distinct module organization between the A-PET− and A-PET+ groups, with reduced network density observed in the A-PET+ group (Figure 4). This finding is consistent with previous findings in the AD cortex, where co-expression networks were disrupted, and in-module connectivity was diminished [45], particularly within pathways associated with astrocytic and microglial anti-inflammatory processes [46]. Likewise, AD biofluids exhibited significant changes in the modules of autophagy, ubiquitination, and sugar metabolism in CSF, as well as in endocytosis and the matrisome in plasma [47]. In the present dataset, module 1 from the A-PET− group was largely disrupted in the A-PET+ group, and this disruption was related to complement activation and innate immune responses. The breakdown of the normal complement and immune network may intensify pro-inflammatory signaling pathways, including NF-κB (Nuclear Factor kappa-light-chain-enhancer of activated B cells) and NLRP3 (NOD-like receptor pyrin domain-containing protein 3) inflammasomes, which are activated by systemic inflammation [48,49]. By overwhelming protective anti-inflammatory responses, these signaling cascades exacerbate microglial dysfunction and impair Aβ clearance, thereby fostering an environment conducive to Aβ oligomerization and systemic accumulation [48,50]. A significant inverse correlation between the module 1 eigengene and OAβ levels in both plasma and Peak A suggests that disruption of immune-related systemic protein networks is associated with increased Aβ oligomerization propensity. Collectively, the results support the hypothesis that alterations in peripheral molecular environments contribute to the regulation of pathogenic Aβ assembly associated with early Alzheimer’s disease-related neural dysfunction.

The expression level of VTN, a hub protein in module 1, was significantly reduced in A-PET+ plasma (Figure 5). VTN is a highly abundant protein in both blood (200–400 μg/mL) and the extracellular matrix [33], and is known to inhibit complement-mediated cytolysis [51] and to participate in cell attachment and migration [52]. Furthermore, VTN also interacts with integrins to regulate endothelial cell homeostasis [29]. VTN-integrin α5 signaling inhibits transcytosis in endothelial cells [53]. Dysregulation of these pathways can result in aberrant angiogenesis and compromise the integrity of the blood–brain barrier (BBB). The exact cause and tissue origin of the reduced VTN remain unclear due to its widespread expression. BBB breakdown is widely recognized as one of the earliest events in AD pathogenesis. Thus, reduced plasma VTN likely reflects an early systemic alteration closely associated with initial vascular disruption. In addition to its vascular functions, recent studies have reported that VTN is involved in neuronal differentiation and neurogenesis [29]. Notably, VTN colocalized with Aβ deposits in the brains of AD patients and inhibited Aβ fibrillization [30,31], underscoring its relevance to AD pathology. Given VTN’s roles in complement regulation and Aβ interaction, reduced systemic VTN levels may create a more permissive environment for pathogenic Aβ assembly in AD. Consistent with this, the addition of recombinant VTN to the plasma of A-PET+ individuals lowered the OAβ levels and suppressed fibril formation. Furthermore, VTN expression levels distinguished A-PET+ individuals (Figure 6). In a multi-stage study, VTN levels declined with AD progression, from preclinical (asymptomatic amyloid-positive) to prodromal AD (mild cognitive impairment with amyloid) [54]. These findings suggest that decreased plasma VTN levels may serve as a biomarker of AD progression and may reflect the transition to early symptomatic AD.

Manipulation of the plasma proteome may modulate OAβ levels and potentially influence disease progression. Multiple studies have demonstrated that plasma exchange enhanced cognitive function in both AD mouse models and aged mice [55,56,57,58]. This bidirectional communication suggests the idea that the albumin interactome serves as a functional interface between the systemic environment and the brain. The present findings suggest that maintaining a ‘healthy’ albumin interactome, particularly through modulators such as VTN, may promote systemic conditions associated with reduced Aβ aggregation propensity. Together, these results indicate a dynamic interaction between the peripheral plasma environment and the central nervous system, suggesting that plasma composition not only reflects but also influences pathological changes in AD.

Several limitations of this study should be acknowledged. First, the cohort is relatively small, and larger, multi-center studies are required to validate these results. Second, although exogenous VTN reduced OAβ, it did not fully restore the levels to those observed in A-PET− individuals, and plasma VTN concentrations did not completely distinguish A-PET status. These results indicate that additional plasma components contribute to Aβ oligomerization. Third, while VTN was detected in multiple SEC fractions (Peaks A, B, and C), only Peak A showed a robust distinction between the A-PET− and + groups. Further studies are needed to elucidate how VTN and other components of albumin interactome interact to regulate OAβ.

In conclusion, this study not only elucidates the molecular mechanism by which plasma from A-PET+ individuals promotes Aβ oligomerization but also identifies a new diagnostic marker of AD, VTN. The albumin interactome plays a pivotal role in modulating OAβ, with Peak A showing systemic changes in both protein composition and co-expression network structure among A-PET+ individuals, particularly within network module 1. VTN inhibits Aβ oligomerization and fibrilization, suggesting that it acts as an endogenous modulator of pathogenic Aβ assembly. Decreased VTN in plasma from A-PET+ individuals may contribute to enhanced OAβ and serve as a potential diagnostic marker of AD. These results highlight the interaction between peripheral AD biomarkers and AD pathophysiology and provide a framework for understanding how systemic proteomic environments may interact with AD-related neural dysfunction. Future longitudinal studies with larger, diverse cohorts and in vivo models are needed to fully assess the therapeutic potential of targeting the albumin interactome and VTN in AD.

## 4. Materials and Methods

### 4.1. Subjects

Plasma samples were obtained from a prospective cohort of community-dwelling older adults recruited at Yongin Severance Hospital (Yongin-si, Republic of Korea) through community advertisement between September 2020 and April 2021. Eligible participants were 65–79 years of age, literate, and had hearing and vision sufficient to complete neuropsychological testing, with a normal range of activities of daily living. Individuals were excluded if they had a history of major neurological disease (e.g., Parkinsonism, epilepsy, stroke, or head trauma), major psychiatric disorder (e.g., schizophrenia, bipolar disorder, or major depression), alcohol or substance use disorder, severe physical illness, abnormal brain MRI findings (e.g., hemorrhage, infarction, or other space-occupying lesions), or contraindications to MRI.

Objective cognitive function was assessed using the Seoul Neuropsychological Screening Battery-Core (SNSB-C), which evaluates five cognitive domains (attention, language, visuospatial function, memory, and frontal/executive function) and yields composite Z-scores adjusted for age, sex, and education. Participants who met the National Institute on Aging–Alzheimer’s Association (NIA-AA) criteria for mild cognitive impairment (MCI) or dementia, based on the SNSB-C and clinical evaluation, were classified accordingly, whereas those who did not were regarded as having objectively normal cognition. Among the cognitively normal participants (CN), subjective cognitive decline (SCD) was operationally defined using two validated self-report scales—the Subjective Cognitive Decline Questionnaire (SCD-Q; 24 items, range 0–24) and the Memory Age-associated Complaint Questionnaire (MAC-Q; 6 items)—with participants scoring SCD-Q ≥ 7 and MAC-Q ≥ 25 assigned to the SCD group and the remainder to the CN group.

All participants underwent amyloid PET imaging with [^18^F] flutemetamol. Each scan was classified as amyloid-positive (A-PET+) or amyloid-negative (A-PET−) by visual read, following the manufacturer’s approved visual interpretation criteria for [^18^F] flutemetamol (representative PET images are in Supplementary Figure S4). Visual interpretation was performed independently by two appropriately trained readers, a nuclear medicine physician and a geriatric psychiatrist, and any discrepancies between the two readers were resolved by consensus. Apolipoprotein E (APOE) genotype was determined by polymerase chain reaction, and carriers of at least one ε4 allele were classified as APOE4-positive.

From this cohort, 141 participants were enrolled, of whom 33 were randomly selected for the present study. This study was approved by the Institutional Review Board of Yongin Severance Hospital (No. 9-2020-0080), and all participants provided written informed consent. Demographic Information and APOE genotype distribution are described in Supplementary Table S1 for Figure 1B, Supplementary Table S2 for Figure 2 and Figure 6F, Supplementary Table S3 for Figure 3, Figure 4 and Figure 5, and Supplementary Table S4 for Figure 6B. The A-PET− and A-PET+ groups were clinically matched to isolate the specific effects of cerebral amyloid pathology. There were no significant differences in the distribution of clinical diagnoses (CN, SCD, MCI) or average CDR-SB scores between the two groups. This design ensures that the observed proteomic alterations are primarily driven by amyloid status rather than variations in cognitive decline.

### 4.2. Preparation of Aβ Monomer, Oligomer, Fibril

Aβ peptide preparations were performed as previously described [59]. Briefly, Hexafluoroisopropanol (HFIP)-treated Aβ was dissolved in DMSO to a 5 mM stock. Monomeric Aβ was prepared by diluting the DMSO stock in PBS to 100 μM immediately prior to use. Oligomeric Aβ was obtained by diluting the stock in ice-cold PBS to 100 μM, then incubating at 4 °C for 24 h. Fibrillar Aβ was prepared by diluting the Aβ stock in 10 mM HCl to 100 μM and incubating at 37 °C for 24 h. The aggregation states of each preparation were verified by Western blot analysis.

### 4.3. Measurement of Oligomeric Aβ Tendency (MDS-OAβ)

OAβ levels were measured using the AlzOn™ assay kit (PeopleBio, Inc., Seongnam-si, Republic of Korea). This assay is based on the MDS, a modified ELISA that employs epitope-overlapping antibodies specific to the N-terminus of Aβ for selective detection of oligomeric Aβ. The assay was performed according to the manufacturer’s protocol. Briefly, PBR-1 (a synthetic Aβ peptide, PeopleBio, Inc., Republic of Korea) was spiked into plasma samples and incubated at 37 °C for 48 h. The PBR-1 mixture and serially diluted standard Aβ oligomer samples were added to a plate coated with the 6E10 antibody and incubated for 1 h at room temperature (RT). After three washes with the washing buffer, the WO2-HRP detection antibody was added and incubated for 1 h at RT. Luminescence was measured using a CLARIOstar Plus reader (BMG LABTECH, Ortenberg, Germany).

### 4.4. Depletion of Plasma Proteins

Selective depletion of major plasma proteins was performed using commercial affinity-based depletion kits according to the manufacturers’ protocols. Depletion efficiency was verified by sodium dodecyl sulfate-polyacrylamide gel electrophoresis (SDS-PAGE) comparing raw plasma and depleted plasma, followed by densitometric quantification using ImageJ (v. 1.54; National Institutes of Health, Bethesda, MD, USA).

#### 4.4.1. Depletion of High-Abundance Plasma Proteins

Fourteen abundant plasma proteins (albumin, IgA, IgD, IgE, IgG, IgG (light chains), IgM, alpha-1-acid glycoprotein, alpha-1-antitrypsin, alpha-1-macroglobulin, apolipoprotein A-I, haptoglobin, transferrin, and fibrinogen) were removed from human plasma using the High-Select™ Top14 Resin (Thermo Fisher Scientific, A36370, Waltham, MA, USA) following the manufacturer’s protocol with minor modifications. Briefly, plasma samples were diluted in PBST and incubated with the resin for 30 min at RT. After incubation, the depleted samples were collected by centrifugation at 1000× *g* for 3 min at RT. The depletion efficiency was confirmed to be 95% (*p* < 0.0001).

#### 4.4.2. Albumin Depletion and Albumin Interactome Extraction

Albumin was depleted from human plasma using the Pierce™ Albumin Depletion Kit (Thermo Fisher Scientific, 85160, USA), following the manufacturer’s protocol with minor modifications. Plasma was mixed with PBST and incubated with the depletion resin for 30 min at RT. The flow-through containing albumin-depleted plasma was collected. The depletion efficiency was confirmed to be 92% (*p* < 0.0001). The albumin-bound component (albumin interactome) was eluted from the albumin interactome-bound resin using an elution buffer (250 mM sodium thiocyanate, 20 mM sodium phosphate, pH 7.2). The elution buffer was replaced with PBS using Amicon Ultra-3K centrifugal filters (Merck, UFC500396, Darmstadt, Germany).

#### 4.4.3. Immunoglobulin Depletion from Plasma

Immunoglobulin was removed from plasma using Pierce Protein A/G Agarose (Thermo Fisher Scientific, 20422, USA). As a negative control, Pierce Control Agarose Resin (Thermo Fisher Scientific, 26150, USA) was used. Plasma samples were diluted in PBST and incubated with the agarose resin for overnight at 4 °C. Following incubation, the samples were centrifuged at 1000× *g* for 5 min, and the supernatant containing plasma depleted of immunoglobulin was collected. The depletion efficiency was confirmed to be 85% (*p* < 0.0001).

### 4.5. SEC

Albumin interactome samples were fractionated using a Superdex 200 Increase column (Cytiva, Marlborough, MA, USA) with PBS as the mobile phase at a flow rate of 0.8 mL/min. Fractions were collected based on real-time absorbance profiles, yielding three prominent peaks: Peaks A, B, and C. Collected fractions were concentrated using an Amicon Ultra-3K centrifugal filter (Merck, UFC500396, Darmstadt, Germany). Concentrated samples were analyzed by SDS-PAGE, and gels were stained with Coomassie Brilliant Blue R-250 (Bio-Rad, 1610437, Hercules, CA, USA).

### 4.6. Liquid Chromatography–Tandem Mass Spectrometry (LC-MS/MS)

SDS-PAGE separated Peak A samples, and gel pieces were subjected to in-gel trypsin digestion. The resulting peptides were extracted and analyzed by LC-MS/MS (Proteinworks, Inc., Daejeon-si, Republic of Korea). Peptides were separated using an analytical column (PepMap RSLC C18, 2 µm, 100 Å, 75 µm × 25 cm; Thermo Fisher Scientific, ES902) coupled to a Q Exactive Plus mass spectrometer (Thermo Fisher Scientific) operating in positive ion mode (ESI+). Full MS scans were acquired at a resolution of 70,000 (AGC target 1 × 10^6^, scan range 350–1900 *m*/*z*), followed by dd-MS2 (HCD, 27%) at a resolution of 17,500 (AGC target 5 × 10^4^, isolation window 1.5 *m*/*z*). Mass spectra were searched against the reviewed *Homo sapiens* protein database (UniProt, taxonomy ID: 9606) using Proteome Discoverer software (version 2.5) with the Sequest HT and Percolator algorithms. The search parameters were set as follows: trypsin digestion with a maximum of two missed cleavages, carbamidomethylation of cysteine as a fixed modification, oxidation of methionine as a variable modification, and mass tolerances of ±10 ppm for precursor ions and ±0.02 Da for fragment ions. MS QCAL peptide mix (Sigma-Aldrich, MSQC2, St. Louis, MI, USA) was used as an internal standard for normalization. Differentially expressed proteins were determined by paired *t*-test analysis comparing A-PET− and + samples with pairs established based on the experimental sequence.

### 4.7. Gene Ontology (GO) Term Analysis

We performed GO term enrichment analysis on the Peak A proteome identified in both A-PET− and + samples. The quantified proteins were first sorted by their *t*-statistic values obtained from the differential expression analysis. Before the enrichment testing, protein lists were filtered to exclude immunoglobulins. For each GO term, enrichment or depletion was assessed using the area under the receiver operating characteristic curve (AUROC) within the protein ranking, where AUROC values greater than 0.5 indicated enrichment and values less than 0.5 indicated depletion. The GO biological process was obtained from the GO.db and org.Hs.eg.db packages in R (v.4.2.1).

### 4.8. Co-Expression Network Analysis

To investigate co-expression patterns and evaluate module preservation between A-PET− and + groups, we performed WGCNA using the WGCNA R package (v1.72-1). Protein expression matrices from plasma samples were transposed, filtered to remove proteins with zero variance or missing values, and assessed for quality using the goodSamplesGenes function of WGCNA.

Unsigned co-expression networks were independently constructed for each group using the blockwiseModules function with a soft-thresholding power of 6, minimum module size of 15, and dynamic tree cut parameters (deepSplit = 3, mergeCutHeight = 0.1). The topological overlap matrix (TOM) was computed to assess protein connectivity strength.

Module preservation between the A-PET− and + groups was evaluated using the modulePreservation function in WGCNA. This method compares network properties, such as density and intramodular connectivity, between the reference (A-PET−) and test (A-PET+) networks, based on 10,000 permutations. Z-summary statistics were used to assess preservation, with modules with Z-summary > 10 considered strongly preserved and those with Z-summary < 2 considered not preserved.

Additional analyses included calculation of module eigengenes, module membership (kME), and intramodular connectivity (kWithin, kTotal) to identify hub proteins. Heatmaps and network graphs were generated using pheatmap, igraph, and ggraph to visualize co-expression structure and module connectivity. All analyses were conducted in R version 4.3.2.

### 4.9. ELISA

The levels of Apo A-II, CD5L, VTN were measured in plasma, albumin interactome and Peak A fractions using the Human Apo A-II ELISA kit (abcam, ab184859, Cambridge, UK), Human CD5L ELISA Kit (Thermo Fisher Scientific, EH94RB, USA), and Human VTN ELISA Kit (Thermo Fisher Scientific, EHVTN, USA) according to the manufacturer’s instructions. Plasma samples were diluted in assay buffer at 1:50,000 for Apo A-II, 1:40,000 for CD5L, and 1:20,000 for VTN. Albumin interactome samples were diluted 1:25,000 for Apo A-II, 1:5000 for CD5L, and 1:50 for VTN. Peak A fractions were diluted at 1:50 for Apo A-II, 1:2000 for CD5L, and 1:10 for VTN in assay buffer. For Apo A-II quantification, samples and recombinant standards were incubated with a cocktail of capture and detector antibodies in antibody diluent for 1 h at RT. After three washes, TMB substrate was added for color development. For CD5L and VTN, samples and standards were incubated overnight at 4 °C. After three washes, detection antibodies and streptavidin-HRP were applied, followed by TMB substrate. Absorbance was measured at 450 nm, and concentrations were calculated using linear standard curves.

### 4.10. Thioflavin T (ThT) Assay

Briefly, 2.5 µM Aβ (PeopleBio, Republic of Korea) was mixed with various concentrations of VTN (Gibco, AF-140-09, Grand Island, NE, USA) in the presence of 20 µM ThT in PBS. The reaction mixture was loaded into a 96-well microplate (165305, Thermo Fisher Scientific, EHVTN, USA) and incubated at 37 °C with shaking at 300 rpm for 42 h. Fluorescence was measured every 10 min using a CLARIOstar Plus reader (BMG LABTECH, Germany) with an excitation wavelength of 450 nm and an emission wavelength of 510 nm.

### 4.11. Pull-Down Assay

PBR-1 was conjugated to Tosyl-activated magnetic beads (Invitrogen, M-280, Carlsbad, CA, USA) according to the manufacturer’s instructions. PBR-1-conjugated beads were incubated with or without VTN in PBST for 1 h with end-over-end rotation at 37 °C. Then, the beads were eluted by boiling at 70 °C for 10 min in 2X Laemmli sample buffer (Bio-Rad, 1610737, USA). The eluted proteins were analyzed by Western blot using the following antibodies: rabbit anti-VTN antibody (Invitrogen, MA5-32157; 1:1000), anti-Aβ antibody (6E10; BioLegend, San Diego, CA, USA, 803003; 1:2000), anti-rabbit IgG-HRP (Sigma, A6154; 1:10,000), and anti-mouse IgG-HRP (Sigma, A9044; 1:10,000). Signal detection was performed using enhanced chemiluminescence reagent (Thermo Fisher Scientific, 34095, USA), and blots were visualized using the ImageQuant™ 800 system (Cytiva, USA).

### 4.12. In Silico Protein Binding Modeling

The binding structure between VTN and PBR-1 was predicted via Protenix (AlphaFold3) analysis (https://neurosnap.ai/service/Protenix%20%28AlphaFold3%29 (accessed on 7 March 2025)). Structure prediction was conducted with the following settings: Template mode = none, MSA mode = mmseqs2_uniref_env, Pair Mode = unpaired_paired, Number Recycle = 6, Recycle Early Stop Tolerance = 0.75, and Number Ensembles = 1. The amino acid sequence of human VTN was retrieved from UniProt (P04004). The Aβ sequence used was identical to the synthetic Aβ (PBR-1) employed in the MDS-OAβ assay.

### 4.13. Equipment Information

Western blot and SDS-PAGE gel images were acquired using the Amersham ImageQuant™ 800 biomolecular imager (Cytiva, USA). Image acquisition and export were performed using Amersham ImageQuant 800 Control Software V2.0.1 (Cytiva). Raw images were exported in TIFF format without selective brightness or contrast adjustment. No selective enhancement, deletion, or manipulation was applied to the original images.

### 4.14. Statistical Analysis

Statistical analyses were performed using R version 4.3 [60] or GraphPad Prism (version 9.5.1; GraphPad Software, Boston, MA, USA). Data were analyzed using Student’s *t*-test, one-way or two-way ANOVA, followed by Tukey’s post hoc test for multiple comparisons. Outliers were identified using Grubbs’ test (*p* < 0.05) and excluded.

## Data Availability

The minimal dataset supporting the conclusions of this article was uploaded with the submission and is available in the Supplementary Materials.

## Supplementary Materials

The following supporting information can be downloaded at: https://www.mdpi.com/article/10.3390/ijms27135744/s1.

## Abbreviations

The following abbreviations are used in this manuscript:

Aβ	Amyloid beta
AD	Alzheimer’s disease
A-PET	Amyloid PET
Apo A-II	Apolipoprotein A-II
APOE	Apolipoprotein E
CD5L	CD5 antigen-like
ELISA	Enzyme-linked immunosorbent assay
FCN1	Ficolin-1
GO	Gene Ontology
H4C1	Histone H4
HFIP	Hexafluoroisopropanol
INHBE	Inhibin beta E chain
LC–MS/MS	Liquid chromatography–tandem mass spectrometry
LDHA	Lactate dehydrogenase A
MDS	Multimer Detection System
NF-κB	Nuclear Factor kappa-light-chain-enhancer of activated B cells
NLRP3	NOD-like receptor pyrin domain-containing protein 3
OAβ	Aβ oligomerization propensity
PET	Positron emission tomography
SDS-PAGE	Sodium Dodecyl Sulfate Polyacrylamide Gel Electrophoresis
SEC	Size-exclusion chromatography
SV2A	Synaptic vesicle glycoprotein 2A
TGF-β	Transforming Growth Factor-Beta
ThT	Thioflavin T
VTN	Vitronectin
WGCNA	Weighted gene co-expression network analysis
