# Supplementary material for: Vitronectin Modulates Plasma Aβ Oligomerization Propensity Within Altered Albumin Interactome Networks in Alzheimer’s Disease
Source: Int J Mol Sci. 2026 Jun 25;27(13):5744. doi: 10.3390/ijms27135744 (PMC13362318; doi:10.3390/ijms27135744)
Supplement: Supplementary file 1 [file ijms-27-05744-s001.zip › Figure S1.pdf]

Supplementary Figure S1. Profile of size exclusion chromatography (SEC) analyzing albuminome from A-PET- and + individuals.

A

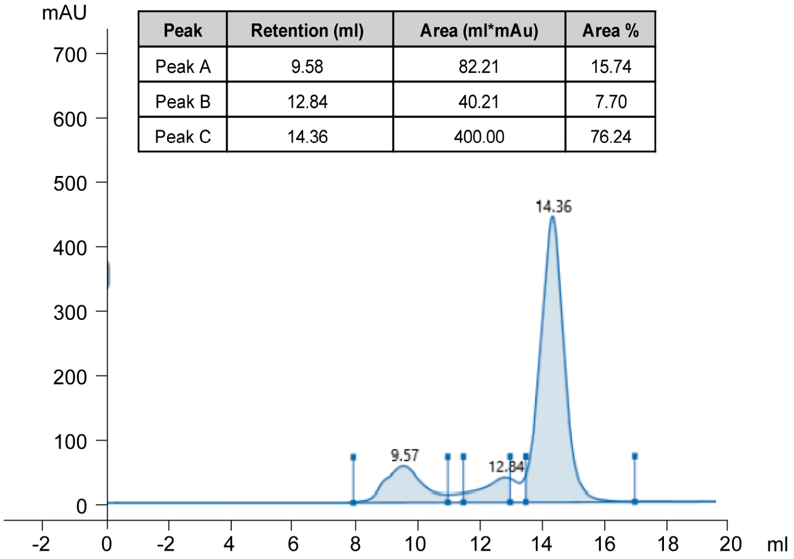

Peak	Retention (ml)	Area (ml*mAu)	Area %
Peak A	9.58	82.21	15.74
Peak B	12.84	40.21	7.70
Peak C	14.36	400.00	76.24

B

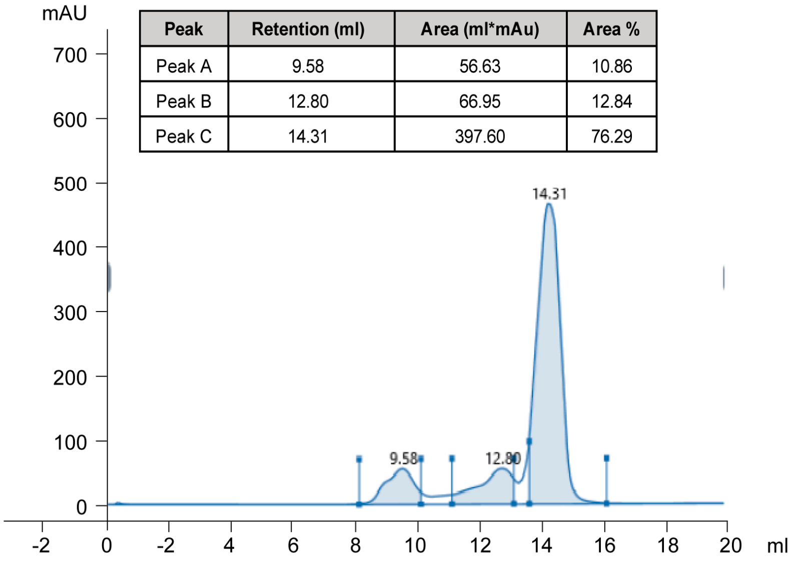

Peak	Retention (ml)	Area (ml*mAu)	Area %
Peak A	9.58	56.63	10.86
Peak B	12.80	66.95	12.84
Peak C	14.31	397.60	76.29

A. Size-exclusion chromatography (SEC) profiles of albuminome samples from A-PET- and **B.** A-PET+ groups, monitored by UV absorbance at 280 nm. Albuminome SEC peaks show Peak A, B, and C. These peaks approximately corresponded to the following molecular weight ranges: Peak A (440-660 kDa or higher), Peak B (70-440 kDa), and Peak C (30–70 kDa).
