# Supplementary material for: Vitronectin Modulates Plasma Aβ Oligomerization Propensity Within Altered Albumin Interactome Networks in Alzheimer’s Disease
Source: Int J Mol Sci. 2026 Jun 25;27(13):5744. doi: 10.3390/ijms27135744 (PMC13362318; doi:10.3390/ijms27135744)
Supplement: Supplementary file 1 [file ijms-27-05744-s001.zip › Figure S2.pdf]

Supplementary Figure S2. Co-expression network visualization of A-PET- and + individuals.

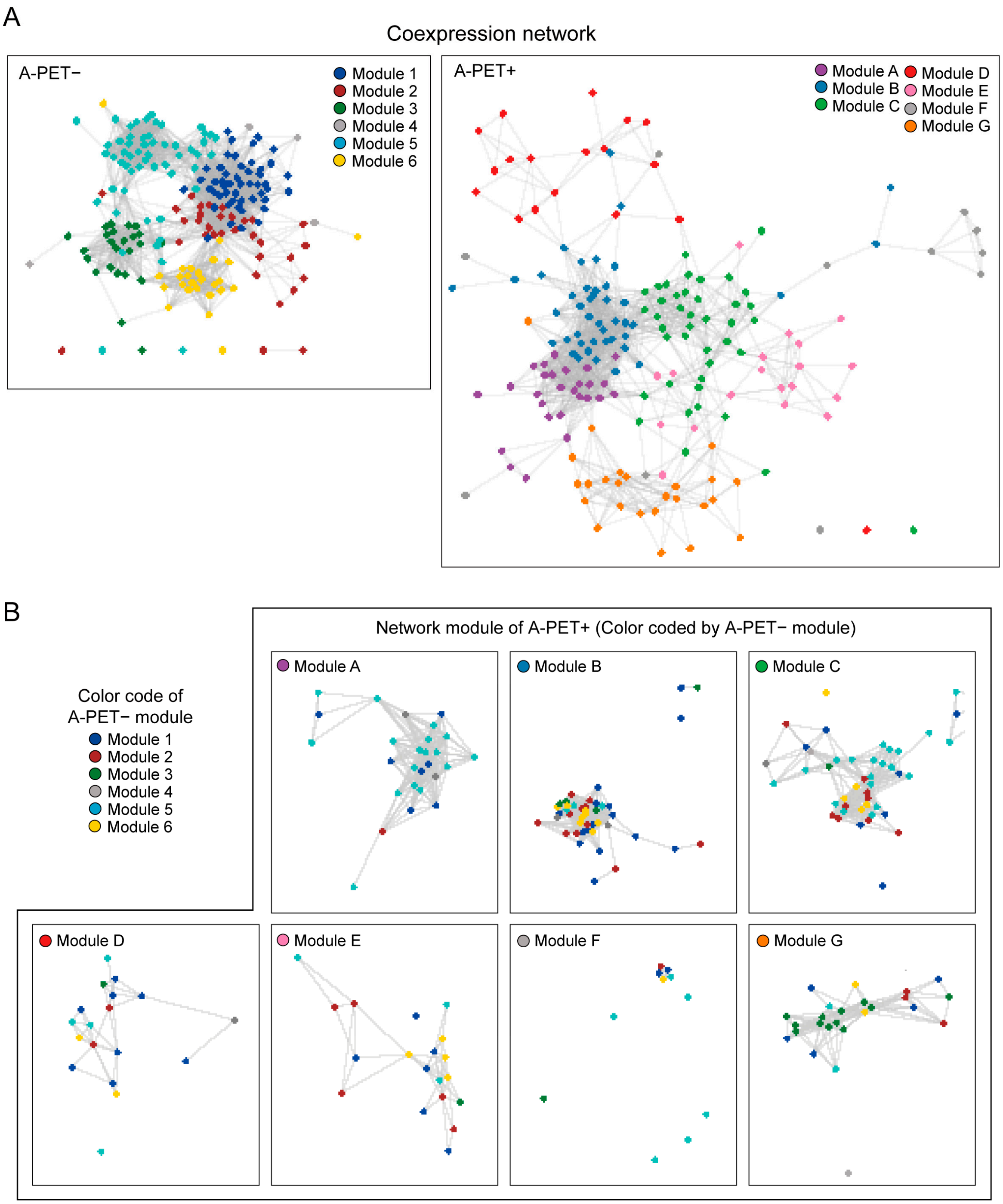

A. Co-expression networks of A-PET- (left panel) and + (right panel) groups. **B.** Co-expression network module of A-PET+ by color code of A-PET- modules.
