# Supplementary material for: Vitronectin Modulates Plasma Aβ Oligomerization Propensity Within Altered Albumin Interactome Networks in Alzheimer’s Disease
Source: Int J Mol Sci. 2026 Jun 25;27(13):5744. doi: 10.3390/ijms27135744 (PMC13362318; doi:10.3390/ijms27135744)
Supplement: Supplementary file 1 [file ijms-27-05744-s001.zip › Figure S3.pdf]

Supplementary Figure S3. CD5L did not alter OAβ.

A

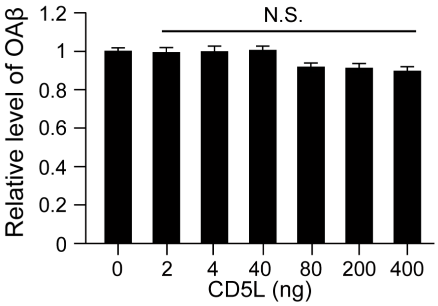

The relative level of OAβ with indicated amount of CD5L introduction (0, 2, 4, 40, 80, 200, 400 ng). Statistical significance was determined by One-way ANOVA with Turkey's post hoc test. Data are expressed as mean ± SEM.
