# Supplementary material for: Vitronectin Modulates Plasma Aβ Oligomerization Propensity Within Altered Albumin Interactome Networks in Alzheimer’s Disease
Source: Int J Mol Sci. 2026 Jun 25;27(13):5744. doi: 10.3390/ijms27135744 (PMC13362318; doi:10.3390/ijms27135744)
Supplement: Supplementary file 1 [file ijms-27-05744-s001.zip › Figure S4.pdf]

Supplementary Figure S4. Representative amyloid PET images acquired with [18F]-Flutemetamol.

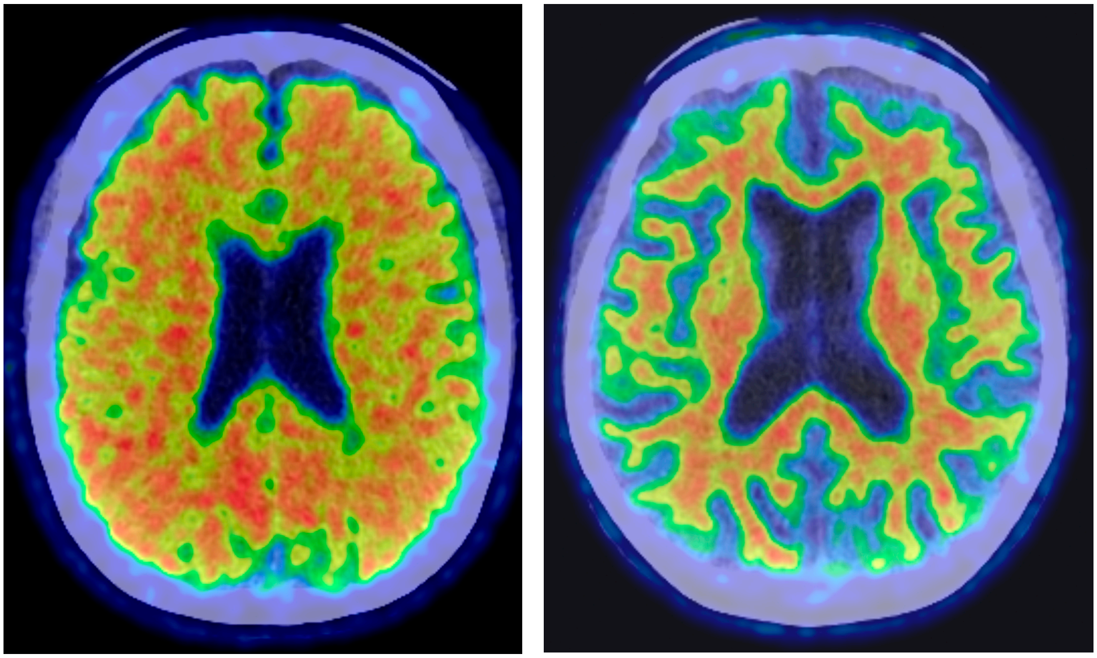

Representative scans classified as amyloid-positive (left) and amyloid-negative (right) based on visual assessment.
